# P-280. HIV and Hepatitis B Virus (HBV) Co-infection in a Multicenter Cohort Study

**DOI:** 10.1093/ofid/ofaf695.501

**Published:** 2026-01-11

**Authors:** Maria F Sanes Guevara, Ken Ho, Anandi N Sheth, Andrew Edmonds, Audrey L French, David B Hanna, Heather King, Jennifer C Price, Maria L Alcaide, Matthew J Mimiaga, Michael Augenbraun, Michael Plankey, Valentina Stosor, Bernard J C Macatangay, Chloe Thio, Yijia Li

**Affiliations:** University of Pittsburgh, Pittsburgh, PA; University of Pittsburgh, Pittsburgh, PA; Emory University School of Medicine, Atlanta, Georgia; The University of North Carolina at Chapel Hill, Chapel Hill, North Carolina; Stroger Hospital of Cook County, Chicago, Illinois; Albert Einstein College of Medicine, Bronx, New York; University of Mississippi Medical Center, Jackson, Mississippi; University of California, San Francisco, San Francisco, CA; Division of Infectious Diseases, Department of Medicine, University of Miami Miller School of Medicine, Miami, FL; UCLA, Los Angeles, California; SUNY Downstate Medical University, Brooklyn, NY; Georgetown University, Washington, District of Columbia; Northwestern University Feinberg School of Medicine, Chicago, Illinois; University of Pittsburgh, Pittsburgh, PA; Johns Hopkins, Baltimore, MD; University of Pittsburgh, Pittsburgh, PA

## Abstract

**Background:**

Hepatitis B virus (HBV) infection disproportionately affects people with HIV (PWH) due to shared transmission routes. Data from the 1990s-2010s showed that 5-10% of PWH had chronic HBV in the United States. In the past decade, broader use of HBV-active and inactive antiretroviral therapy (ART), improved HBV vaccinations, and increasing intravenous drug use (IDU) may have changed the landscape of HBV infection in PWH and people without HIV (PWoH), but this has not been well studied.

**Figure:**
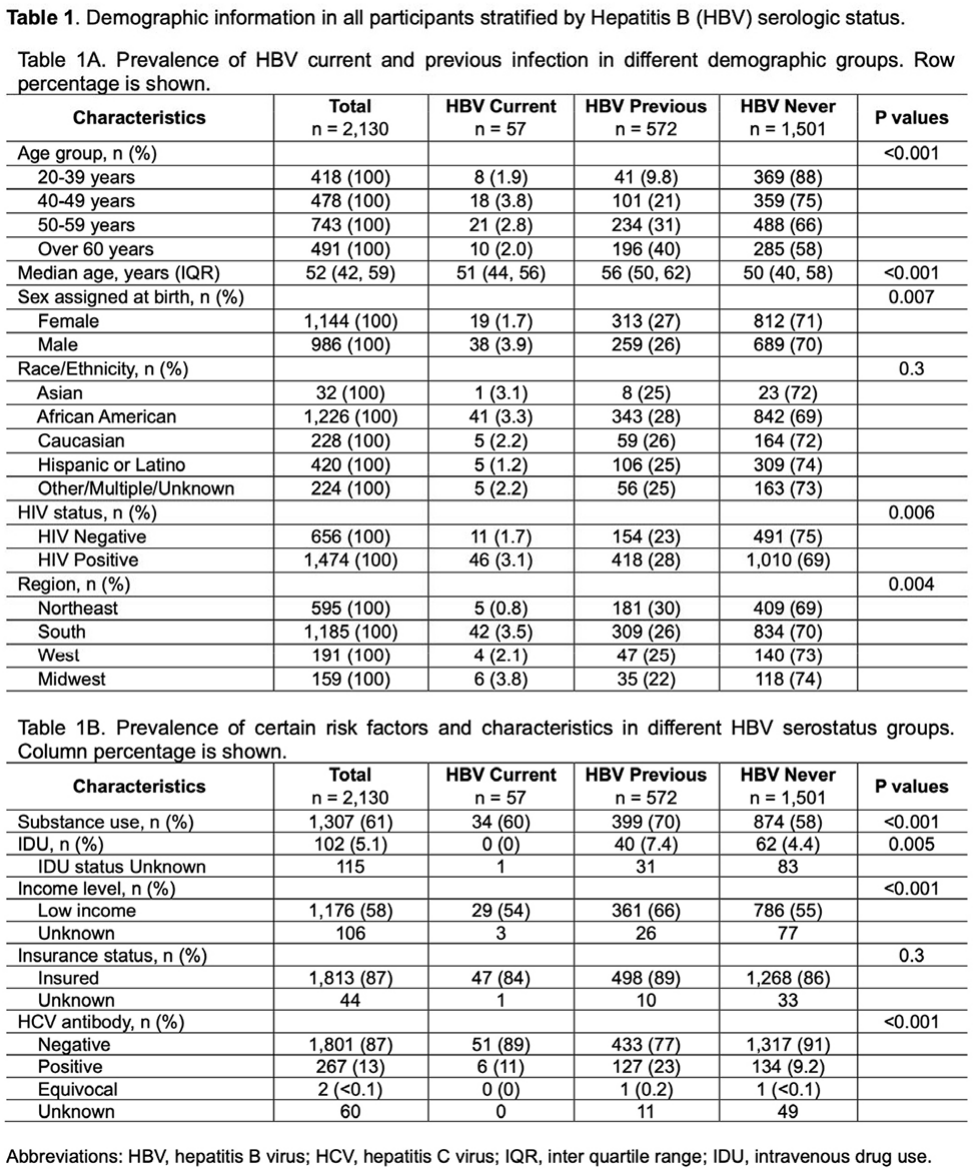

**Figure:**
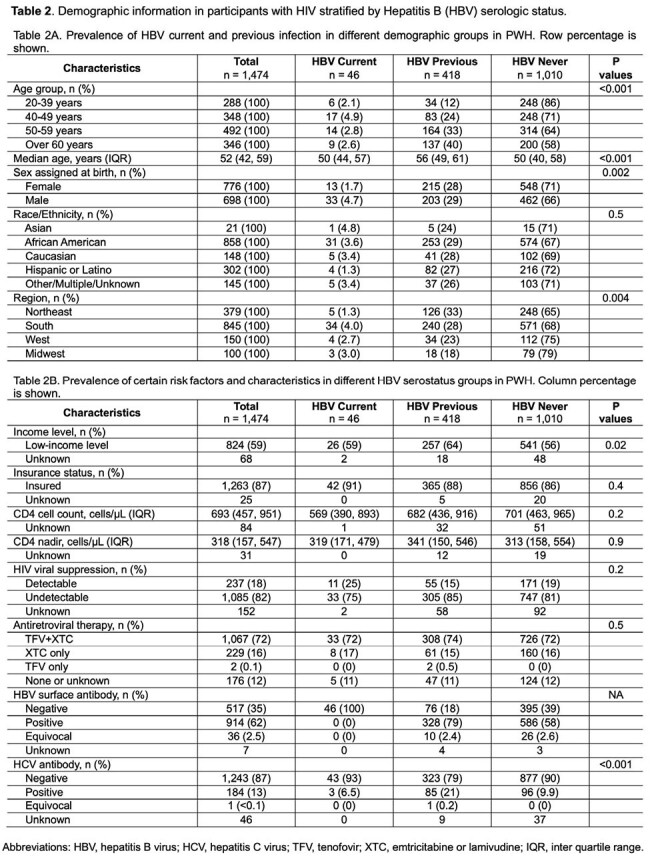

**Methods:**

This study aimed to describe the prevalence of HIV/HBV coinfection among participants enrolled across 13 U.S. MACS/WIHS Combined Cohort Study (MWCCS) clinical research sites. Between 11/2020 and 3/2024, participants underwent serologic testing for HBV. We defined a positive result for HBsAg (surface antigen) as a current infection; negative HBsAg and positive anti-HBV core antibody (anti-HBc) as past infection; and both negative HBsAg and anti-HBc as never infection. We used the Chi-squared or Fisher’s exact test to compare categorical variables and the Kruskal-Wallis test for continuous variables among three HBV serogroups.

**Figure 1:**
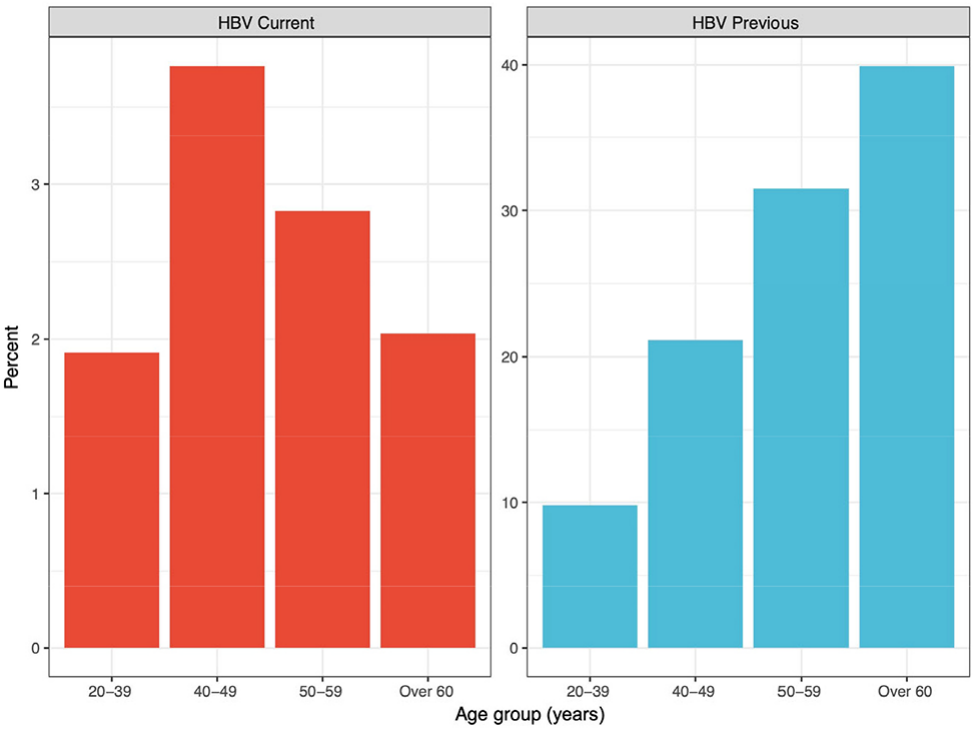
Age group distribution of all participants stratified by Hepatitis B (HBV) serologic status

**Results:**

Among 2130 participants, HIV seropositivity was associated with higher rates of current (PWH 3.1% vs. PWoH 1.7%) and prior HBV infection (PWH 28% vs. PWoH 23%, P=0.006) (Table 1). Current HBV infection was more common in men (3.9% in males and 1.7% in females) and those in the 40-59 age group (3.8% in the 40-49 age group and 2.8% in the 50-59 group) (Figure 1). Prior HBV infection was associated with higher rates of substance use, especially injection drug use, and having been infected with hepatitis C (Table 1B). Among PWH, current HBV infection was more common in men than women (4.7% vs. 1.7%, Table 2A). HIV suppression, CD4 count and nadir, and HBV-active ART use were similar among PWH across HBV categories (Table 2B). In PWH who never had HBV infection, 39% did not have immunity against HBV.

**Conclusion:**

The prevalence of HIV/HBV coinfection in this selected group of the MWCCS cohort is 3.1%, lower than previously reported in the 1990s-2010s. Male participants in the age group of 40-59 years are at higher risk for current HBV infection. Our study also highlights the need to screen for HBV immunity in people who never had HBV but are at risk for HBV infection.

**Disclosures:**

Jennifer C. Price, MD, PhD, AbbVie: Grant/Research Support|Gilead: Grant/Research Support|VIR: Grant/Research Support Maria L. Alcaide, MD, Gilead: Advisor/Consultant Valentina Stosor, MD, American Physician Institute: Honoraria|CDC: Grant/Research Support Bernard JC Macatangay, MD, Merck: Grant/Research Support

